# Tonic and burst spinal cord stimulation waveforms for the treatment of chronic, intractable pain: study protocol for a randomized controlled trial

**DOI:** 10.1186/s13063-016-1706-5

**Published:** 2016-12-01

**Authors:** Konstantin V. Slavin, Richard B. North, Timothy R. Deer, Peter Staats, Kristina Davis, Roni Diaz

**Affiliations:** 1 Department of Neurosurgery, University of Illinois College of Medicine, 912 South Wood St, Chicago, IL USA; 2 Departments of Neurosurgery, Anesthesiology and Critical Care Medicine, Johns Hopkins University School of Medicine, Baltimore, MD USA; 3 Center for Pain Relief, 400 Court St #100, South Charleston, WV USA; 4 Premier Pain Centers, 170 Avenue at the Common, Shrewsbury, NJ USA; 5 St. Jude Medical, 6901 Preston Rd., Plano, TX USA

**Keywords:** Neuromodulation, Spinal cord Stimulation, Randomized, Prospective, Comparative efficacy, Burst stimulation, Tonic stimulation

## Abstract

**Background:**

Burst stimulation is a novel form of neurostimulation for the treatment of chronic pain which has demonstrated promise in small uncontrolled studies, but has not yet gained approval for use in the U.S. We report the study methods for an ongoing multicenter, randomized, controlled, cross-over study designed to gain United States Food and Drug Administration (FDA) approval for burst stimulation.

**Methods:**

Participants who are candidates for a currently approved neurostimulation device were enrolled and screened. Participants who fail a tonic trial evaluation, have significant depressive symptoms, or evidence lack of compliance with study procedures by failing to complete 7 days of a Pain Diary are excluded. Participants receiving a permanent implant are randomized to receive: (1) 12 weeks of tonic followed by 12 weeks of burst stimulation or (2) 12 weeks of burst stimulation followed by 12 weeks of tonic stimulation. Assessments occur at 6, 12, 18, and 24 weeks. After 24 weeks, participants choose their preferred therapy and are assessed every 6 months for up to 2 years. All patients had the device leads inserted at the site of a successful tonic stimulation trial. Assessments include: a Pain Diary using a Visual Analog Scale (VAS) for overall, trunk, and limb pain, the Beck Depression Inventory, the Pain Catastrophizing Scale, the Oswestry Disability Index, paresthesia, satisfaction, and therapy preference. Reported adverse events are collected throughout the study. The primary endpoint is the noninferiority of burst stimulation compared to tonic measured by the within-subject difference in the mean overall VAS score at the end of each 12-week stimulation period.

**Discussion:**

This trial represents the largest controlled trial of burst stimulation to date, and is expected to yield important information regarding the safety and efficacy of burst stimulation.

**Trial registration:**

ClinicalTrials.gov, NCT02011893. Registered on 10 December 2013.

**Electronic supplementary material:**

The online version of this article (doi:10.1186/s13063-016-1706-5) contains supplementary material, which is available to authorized users.

## Background

Spinal cord stimulation (SCS) is an established therapy to treat chronic, intractable pain of the trunk and limbs [1]. Traditional SCS produces tonic waveforms in which pulses are delivered at a consistent frequency, pulse width, and amplitude. New technological developments using alternate waveforms to stimulate the dorsal column and/or aim at new stimulation targets show promising results [2]. Burst stimulation, in particular, is a waveform that delivers groups of pulses at a high frequency and at amplitudes much lower than tonic stimulation; these groups of pulses are separated by a pulse-free period called an interburst interval during which passive repolarization occurs prior to the next burst. This pattern was chosen because this burst waveform mimics naturally occurring neuronal firing in the central nervous system [3].

The use of the burst waveform in SCS for the treatment of chronic pain was first reported in 2010 [4]. Since that time, reports from multiple, relatively small, clinical studies have shown that burst stimulation provides effective pain control, which, in some patients, may surpass the pain relief provided by tonic stimulation [5–8]. In these studies, the additional pain reduction during burst stimulation was relatively only marginally smaller compared with tonic stimulation results, but burst stimulation was preferred by a majority of patients in spite of the similar pain-reducing profile. Some evidence indicates that burst stimulation may provide a salvage therapy option for patients who fail to achieve adequate pain control with tonic stimulation or for those in whom the initial efficacy of tonic stimulation is lost over time [7, 8].

A common element in all previous clinical studies is the marked reduction or total absence of paresthesia during burst stimulation. Tonic stimulation is predicated on the use of paresthesiae to “cover” painful areas, and little evidence has been reported for efficacious therapy when tonic stimulation is delivered at subperception levels. Alternatively, burst stimulation appears to produce paresthesia in only a subset of patients, approximately 17–25% [4, 7, 8], and anecdotal reports indicate that paresthesiae during burst stimulation may be qualitatively different from those experienced during tonic stimulation. Perceptual difference in the patient experience may exert an indirect influence on patient-reported outcomes.

To date, the mechanisms and clinical effects of the burst waveform have been mainly studied in small cohorts of patients in Europe and Australia over a short duration (e.g., 2–4 weeks). Larger, longer-term, controlled clinical studies of the burst waveform are lacking. Burst stimulation has not yet been approved for use in the U.S. Here, we report the study methods for an ongoing multicenter, cross-over, randomized controlled study designed to gain United States Food and Drug Administration (FDA) approval for burst stimulation (ClinicalTrials.gov ID: NCT02011893).

## Methods

### Objectives

Under an Investigational Device Exemption (IDE), the objective of the study (protocol C-12-07 ver 8.28.14) is to demonstrate safety and efficacy for burst stimulation. As such, the study examines burst stimulation compared to the traditional SCS stimulation mode (tonic stimulation). The primary objective of the study is to establish noninferiority of pain intensity after 3 months of burst compared to 3 months of tonic stimulation. Previous studies [5–8] indicated that burst stimulation was preferred by most subjects even when the magnitude of pain relief was similar. Therefore, the primary objective of noninferiority of pain intensity will establish the therapy as comparable for pain relief to traditional tonic stimulation, and secondary objectives (listed below) will examine the superiority of pain relief and other outcomes that may elucidate the patient experience of burst stimulation.

Secondary objectives are:

- To demonstrate differences between tonic and burst stimulation in responder rate
- To demonstrate significant differences in the presence of paresthesia during tonic stimulation and burst stimulation
- To demonstrate that pain relief using burst stimulation is superior to pain relief using tonic stimulation
- To demonstrate differences between tonic and burst stimulation adverse events
- To demonstrate differences between tonic and burst stimulation for quality of life
- To demonstrate differences between tonic and burst stimulation for worst pain
- To demonstrate differences between tonic and burst stimulation for function

Additional analyses will examine these objectives after 12 months of therapy.

### Trial design

**Fig. 1:**
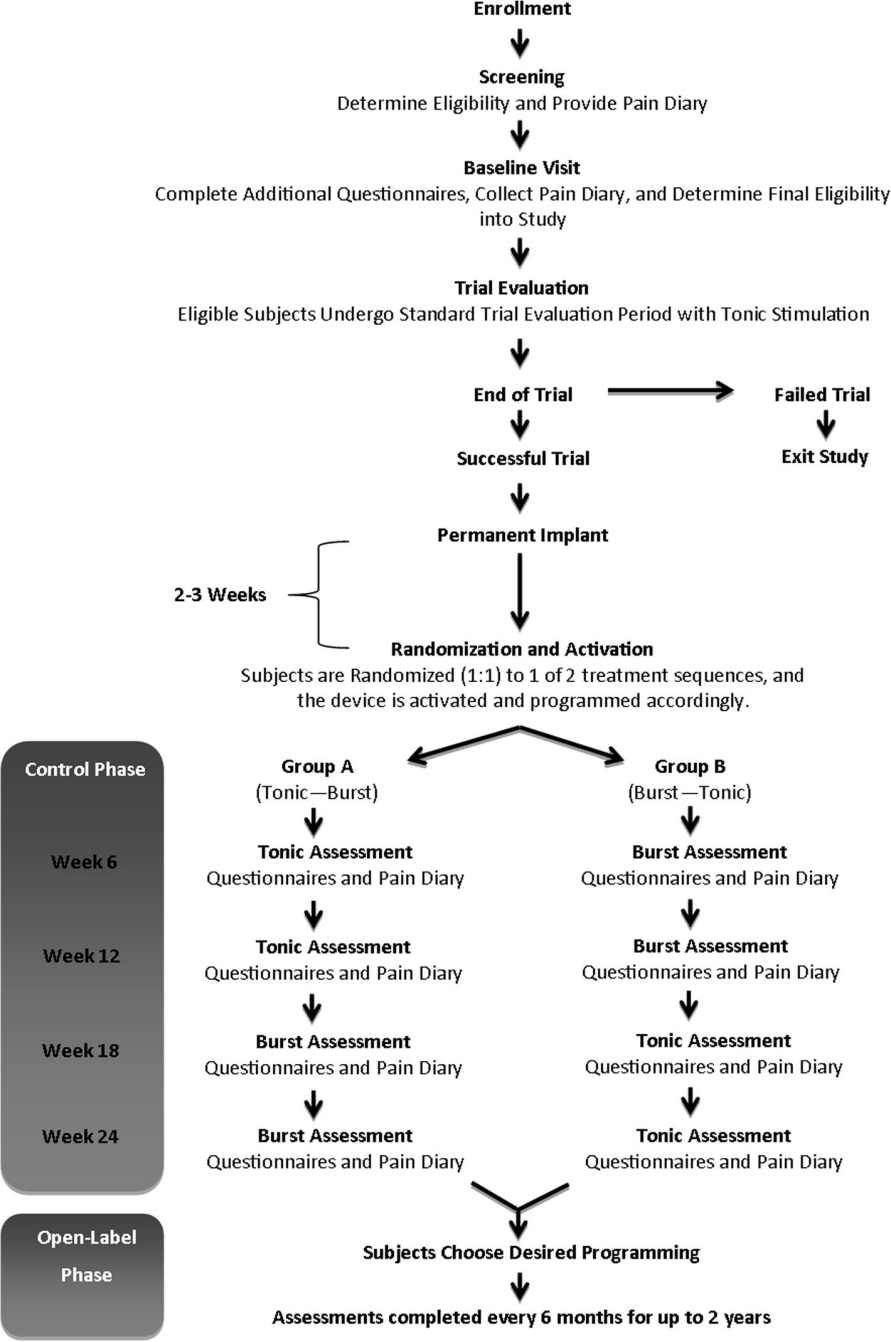
Study design. Schematic of the study design and follow-up schedule

**Fig. 2:**
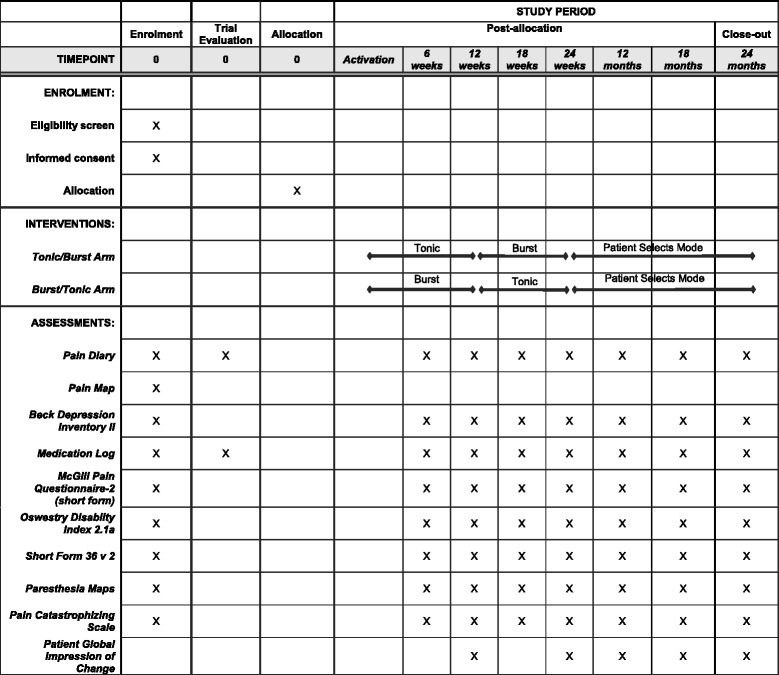
Standard Protocol Items: Recommendations for Interventional Trials (SPIRIT) figure

The study consists of two distinct phases: (1) a 24-week controlled (cross-over) phase during which participants are randomly assigned to one of two treatment sequences of tonic and burst stimulation, each for 12 weeks and (2) an open-label phase during which participants may use either waveform (see Figs. 1 and 2). With guidance from the FDA, the study employs enrichment strategies aimed at decreasing variability and assessing the efficacy of burst compared to an approved SCS waveform. As such, participants are limited to those who achieve adequate pain relief during a traditional tonic stimulation trial evaluation; all participants use both waveforms in a within-subject cross-over design.

At up to 20 centers in the U.S., Institutional Review Board (IRB) approval is obtained (refer to its “Declarations” page), and eligible enrolled participants undergo additional screening and participate in a standard SCS trial evaluation using tonic stimulation. Those with a successful SCS trial evaluation are implanted with the Prodigy™ neurostimulation system (St. Jude Medical, Plano, TX, USA) that can deliver both burst and tonic waveforms. Lead position for the permanent system is determined by placement for optimum paresthesia coverage during the tonic stimulation trial evaluation. After a 2–3 week surgical recovery period, participants with the permanent system are randomized to receive one of the two treatment sequences over the subsequent 24 weeks.

During the control phase, participants use one waveform for 12 weeks then cross over to the other waveform for the remaining 12 weeks. Participants are randomly assigned (1:1) at each study site using computer-generated assignment to receive 12 weeks of tonic stimulation followed by 12 weeks of burst stimulation, or 12 weeks of burst stimulation followed by 12 weeks of tonic stimulation. Participants in both groups complete assessments at 6, 12, 18, and 24 weeks after randomization.

To enhance participant compliance with the study protocol, a washout period was not employed when participants crossed over. This decision was based on previous reports suggesting that 25–80% of patients may fail to comply with the study protocol when asked to undergo periods without stimulation [9, 10]. Assessments occur at a minimum of 6 weeks after changing stimulation modes, which is adequate time to ensure that any after-effects of the previous therapy will not influence the assessment of the current therapy [11, 12].

During the open-label phase of the study, participants attend study visits for assessment every 6 months for up to 2 years post randomization. Perceptible differences in paresthesia between the two waveforms are likely, thus neither patients nor investigators are blinded to treatment sequence.

Any changes to the protocol will be reported to the IRB for each site and to the FDA. The protocol, and this reported version of the protocol conform to the Standard Protocol Items: Recommendations for Interventional Trials (SPIRIT) guidelines; a copy of the SPIRIT Checklist accompanies this report as part of the supplemental materials (see Additional file 1).

### Participants

Participants with chronic neuropathic pain of the trunk and/or limbs are invited to consider study participation. After signing an informed consent, enrolled participants are evaluated for compliance with the inclusion/exclusion criteria (see Table 1). Participants who fail to comply with the Pain Diary assessment (see “Assessments” section) are excluded from the study. Participants who evidence moderate-to-severe depression and/or suicidal ideation, measured by the Beck Depression Inventory II (BDI-II), do not meet the criteria and are excluded from the study. This was derived, with guidance from the FDA, to select participants who were free from serious affective comorbidities requiring behavioral/pharmacological interventions in addition to pain therapy. Participants were prohibited from increasing the dosage of pain medications, with the exception of acetaminophen, during the first 24 weeks of the study. Participants were not required to stop or decrease pain medications at any time during the study.

**Table 1:** Inclusion/exclusion criteria

Inclusion criteria
• Ability to provide informed consent • Aged 22 years or older • Chronic intractable neuropathic pain of the trunk and/or limbs • Baseline average daily overall pain score of ≥60 on the Visual Analog Scale collected with the 7-day Pain Diary • Failed ≥3 documented medically supervised treatments (including, but not limited to, physical therapy, acupuncture, etc.) as well as treatment with ≥2 classes of medication • Stable pain-related medication regimen 4 weeks prior to the screening evaluation • Deemed a good candidate for SCS by the investigator • Deemed a suitable study candidate by a psychologist or psychiatrist • Agrees not to add to or increase pain medication during the study • Willing to cooperate with the study requirements, including compliance with the regimen and completion of all office visits • Female candidates of child-bearing potential agree to commit to the use of an effective method of contraception for the duration of the study
Exclusion criteria
• Current participation in a clinical trial with an active treatment arm • History of a neurostimulation trial or implanted system • Presence of an infusion pump or any implantable neurostimulator • Overall Beck Depression Inventory II score is >24 or, at the screening visit, a score of 3 on Question 9 relating to suicidal thoughts or wishes • Receiving, applying for, or considering workers’ compensation or involved in disability litigation • Concurrent clinically significant or disabling chronic pain problem that requires additional treatment • Existing medical condition that is likely to require repetitive MRI evaluation • Existing medical condition that is likely to require the use of diathermy • History of cancer requiring active treatment in the past 6 months • Pain originating from peripheral vascular disease • Participant is immunocompromised • Documented history of allergic response to titanium or silicone • Documented history of substance (narcotics, alcohol, etc.) abuse or dependency in the 6 months prior to baseline • Pregnancy (confirmed by positive urine/blood test)

*MRI* magnetic resonance imaging, *SCS* spinal cord stimulation

### Interventions

The SCS trial evaluation period, per usual care (approximately 3 to 10 days), is performed with epidural leads placed percutaneously under local anesthesia and connected to an external pulse generator that delivers tonic stimulation (St. Jude Medical, Plano, TX, USA).

The implanted SCS device, Prodigy™ (St. Jude Medical, Plano, TX, USA), a constant current generator, is capable of delivering both tonic and burst waveforms. Surgical implant of the permanent system occurs approximately 4 to 8 weeks after the end of the trial evaluation period, pursuant to usual care and surgical scheduling. After the permanent implant, a surgical recovery period of 2 to 3 weeks occurs to allow for wound healing during which transient medication increases were allowed.

**Fig. 3:**
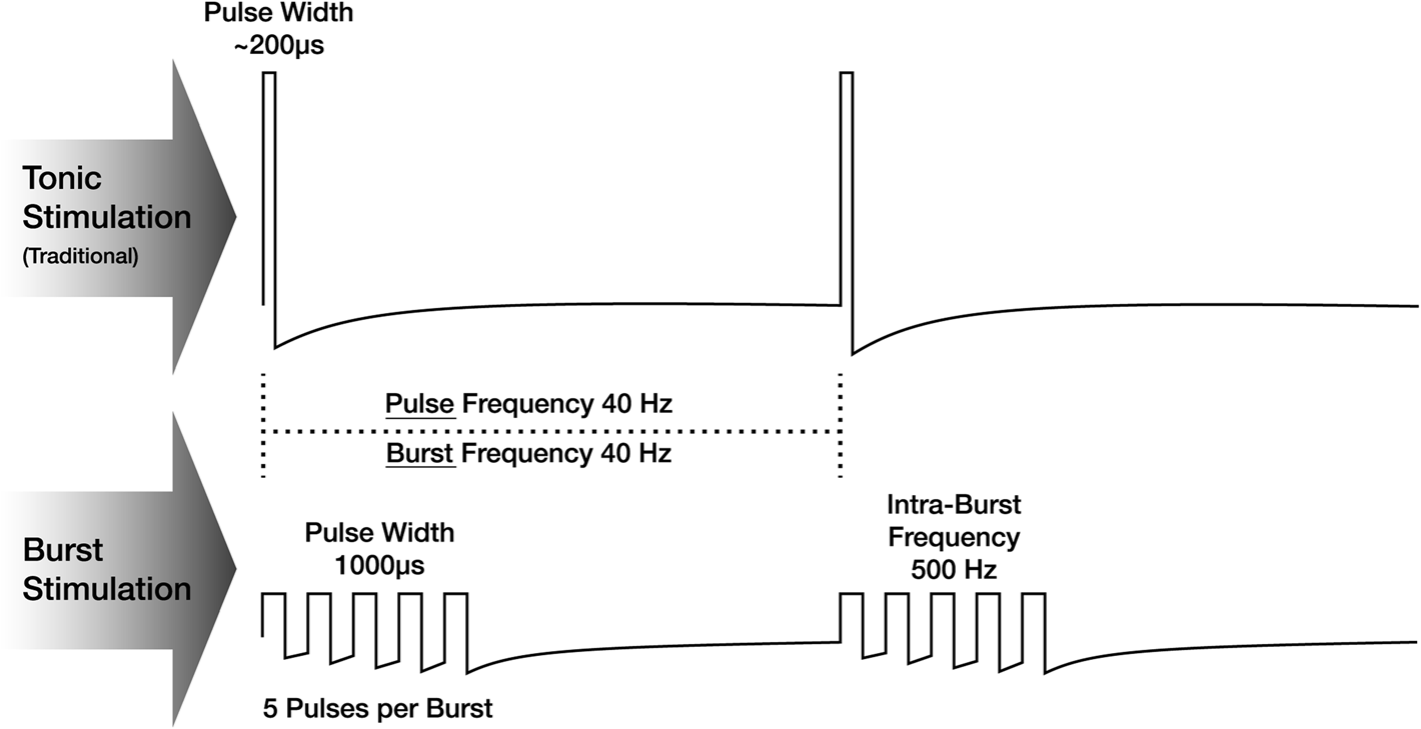
Waveforms. Tonic stimulation provides a consistent stream of pulses at a set frequency, pulse width, and amplitude. Burst stimulation delivers groups of pulses at a lower amplitude and a higher frequency than tonic stimulation. Bursts of pulses are followed by pulse-free periods during which charge balance occurs

Tonic stimulation pulse width is programmed in the usual range of 100–500 μs, and tonic stimulation frequencies are set at between 30 Hz and 100 Hz. Amplitudes for tonic stimulation are programmed according to individual participant perception to a level that typically produces comfortable paresthesia. Burst programming for this study (see Fig. 3) followed specific parameters such that 500-Hz stimulation is delivered in groups of five pulses with 1-ms pulse width, with bursts repeated 40 times per second. Charge balance occurs during the 5 ms after each burst with passive repolarization. Amplitudes for burst stimulation are programmed according to individual participant perception.

During the control phase of the study, participants are advised that they might or might not experience paresthesia at the outset of both waveforms.

### Assessment tools

See Fig. 2 and Table 2 for a schedule of assessments.

**Table 2:** Study visits and associated assessments

Visit	Assessments
Enrollment and screening	• Pain history and demographics • BDI-II • Pain Location Form • Medications • Provide subject with the 7-day Pain Diary for the baseline evaluation
Baseline evaluation	• 7-day Pain Diary returned • SF-MPQ-2 • ODI2.1a • SF-36v2^TM^ • PCS • Medications
Trial system implantation	• 7-day Pain Diary returned • Medications
End of trial visit (4–8 days after trial system implantation)	• Medications • End-of-trial physician assessment
System implantation	• Medications • Physician’s record surgical summary for the procedure, including any complications
Randomization/activation	• Medications • Participant programming record
Control phase follow-up visits (Occurring at 6, 12, 18, and 24 weeks after randomization)	• 7-day Pain Diary returned • SF-MPQ-2 • ODI2.1a • SF-36v2^TM^ • PCS • Medications • Paresthesia mapping • Self-evaluation
End of control phase (Assessments occurring at the 24-week visit, in addition to those described above)	• PGIC • Patient preference
Open-label phase follow-up visits (Occurring every 6 months for up to 2 years)	• 7-day Pain Diary returned • SF-MPQ-2 • ODI2.1a • SF-36v2^TM^ • PCS • Medications • Paresthesia mapping • Self-evaluation

*BDI-II* Beck Depression Inventory BDI-II, *OD12.1a* Oswestry Disability Index 2.1a, *PCS* Pain Catastrophizing Scale, *PGIC* Patient Global Impression of Change, *SF-36v2* ^*TM*^ Short-form 36 Health Survey, *SF-MPQ-2* Short-Form McGill Pain Questionnaire version 2

#### Pain history and participant demographics

Upon enrollment, participants answer relevant questions pertaining to pain history, previous treatment history, and participant demographic information (including height, weight, age, race, and marital status).

#### Pain

A daily Pain Diary is completed for the 7-day period prior to each study visit. In the diary, participants are asked to rate pain intensity using the Visual Analog Scale (VAS), which consists of a horizontal line 100 mm in length, anchored by word descriptors on each end (no pain to worst imaginable pain). Higher scores indicate higher pain levels. Each diary entry includes four different VAS scores to permit calculation of a weekly average for daily overall pain, worst overall pain, trunk pain, and limb pain. Participants arriving at the baseline visit with less than seven diary entries repeat the baseline diary. If repeating the baseline diary results in less than seven entries, the participant is deemed a screening failure. Participants complete the baseline pain diaries without making changes to their current, stable pain medications. After randomization, missing diary entries are accommodated during data analysis (see “Statistical methods” section for more details).

Participants also complete the Short-Form McGill Pain Questionnaire version 2 (SF-MPQ-2), a validated questionnaire that measures the qualities of pain [13]. The SF-MPQ-2 is a self-administered questionnaire consisting of 22 different pain descriptors. The participant indicates the intensity of each pain descriptor as it pertains to the participant’s pain experience. Intensity ratings for each descriptor are provided on a scale ranging from 0 (“none”) to 10 (“worst possible”). The total SF-MPQ-2 score is the sum of the score of three sensory subscales (continuous, intermittent, and predominantly neuropathic pain descriptors) and one affective subscale. Higher scores indicate a higher severity of symptoms.

#### Medication

Throughout the study, site study personnel complete a medication log to track the participants’ reported daily doses and names of pain medications (prescription and over-the-counter) and medications known to affect pain perception (e.g., certain antidepressants).

#### Quality of life

The SF-36v2™ Health Survey [14] is a validated, self-administered short-form health questionnaire with 36 questions. Scoring produces eight subscale scores for vitality, physical functioning, bodily pain, general health perceptions, physical role functioning, emotional role functioning, social role functioning, and mental health. These eight subscales are used to calculate two summary component scores for physical and mental health. Higher scores indicate a better quality of life.

#### Pain catastrophizing

The Pain Catastrophizing Scale (PCS) is a validated scale to assess the quality of thoughts and feelings during pain [15]. The PCS includes 13 statements for which participants rate the frequency with which the statement matches their pain experience, from 0 (“not at all”) to 4 (“always”). The scale is self-administered, takes 5 min to complete, and examines three domains: rumination, magnification, and helplessness. A higher score indicates a higher level of catastrophizing.

#### Disability

The Oswestry Disability Index 2.1a (ODI) is a validated low-back pain questionnaire that indicates the extent to which a person’s functional level is restricted by disability [16]. The self-administered questionnaire includes 10 questions and takes approximately 5 min to complete. The questionnaire produces a percentage total score that indicates the degree of disability; a higher percentage indicates a greater disability.

#### Mood

The Beck Depression Inventory BDI-II is a 21-question validated assessment that evaluates the intensity of depressive symptoms [17]. The questionnaire takes approximately 5 min to complete. The total score is categorized by minimal, mild, moderate, and severe depression. Higher total scores indicate more severe depressive symptoms.

#### Pain and paresthesia mapping

Pain location is documented on a map of the body labeled with different numbered segments. The participant is instructed to shade-in or place an “X” in the area(s) of pain.

Paresthesia mapping is documented using a body map similar to that used for pain location mapping. The participant is instructed to shade-in or place an “X” in the area(s) for which they experience paresthesia or other perceptible stimulation sensations. Participants are also asked to indicate the area or areas on the body map for which pain relief translates to the greatest improvement for their daily activities.

#### Patient Global Impression of Change

At the 12-and 24-week visits and at all long-term follow-up visits, participants are asked to complete a standard seven-item Patient Global Impression of Change (PGIC) Likert scale, ranging from “no change” to “a great deal better.”

#### Therapy preference and satisfaction

After using both therapy types (at 24 weeks), participants are asked to indicate which waveform they prefer and to provide a reason for their preference.

At each follow-up, participants are asked to rate satisfaction with the device by selecting from the following options on a 5-point Likert scale: very satisfied, satisfied, neither satisfied nor dissatisfied, dissatisfied, or very dissatisfied.

#### Adverse events

Adverse events (AEs) are recorded for enrolled participants at any visit, including unscheduled visits. AEs are classified according to their relationship to the device(s) and/or procedures and according to severity. All AEs, regardless of the reason or severity, are followed with appropriate corrective actions by the investigators until a satisfactory resolution is obtained.

### Study visits

Table 2 shows the assessments scheduled for each study visit. At the enrollment visit, participants adhering to the initial inclusion/exclusion criteria sign informed consent, complete assessments, and start the VAS Pain Diary. If required of female participants, a pregnancy test is performed. Participants who meet all the inclusion/exclusion criteria after initial assessments and completion of the baseline Pain Diary complete additional assessments and are scheduled for the tonic stimulation trial evaluation period for 3–7 days. Participants with a successful trial, defined as a patient-reported pain reduction of at least 50%, proceed to a permanent implant. After a surgical recovery period of 2–3 weeks, participants are randomized (1:1 at each site) using an online electronic program provided by St. Jude Medical and the prescribed programming is activated. All participants receive a patient programmer that enables activation of the ON and OFF positions and adjustments to amplitude within the prescribed range.

During the control phase of the study, participants attend follow-up visits at the mid-point and end of each of the two 12-week phases of treatment. At the 12-week visit, participants cross over to the second stimulation mode of their treatment sequence and continue in the study for an additional 12 weeks (through 24 weeks).

Participants are programmed to their preferred stimulation mode at the end of the 24-week visit. Subsequently, participants attend follow-up visits every 6 months for up to 2 years. During this open-label phase, participants can request reprogramming and may use either stimulation mode or both, as needed to maintain adequate therapeutic benefit.

### Statistical methods and data management

Data management, including monitoring, is performed by the study sponsor to ensure data completeness and accuracy. Additionally, a noninvestigator physician Steering Committee performs data review and adjudication. As part of standard practice for IDE studies, the FDA conducts occasional audits of the study conduct, data collection, and data management. Study investigators will have access to the data after study closure and will be offered the opportunity to participate as authors on publications. Electronic data capture with appropriate security protocols is used to ensure participant confidentiality during and after the study closure.

Statistical analyses will be performed using a significance level of *p* = 0.05, unless otherwise specified, and 95% confidence intervals will be computed. Interim analyses for the primary endpoint will occur when all randomized participants complete the 24-week visit; results of that analysis will be submitted for publication. Analyses of the long-term follow-ups will be reported after study closure.

#### Sample size

Noninferiority of the VAS score during burst compared with tonic stimulation was assumed to be within a window of 7.5 mm on the 100-mm VAS. Based on earlier reports, the standard deviation of the difference between tonic and burst stimulation VAS scores was assumed to be 18.4 mm. With a type I error rate of 0.05, a minimum sample of 76 participants was required to achieve 80% power to show noninferiority between the two stimulation modes.

Previous studies indicate that approximately 29% of subjects trialed with tonic stimulation do not receive a permanent implant and an additional 20% of subjects may be lost to attrition by the 6-month visit [18]. Screen failure rates were originally estimated to be at 15%, but after the first 6 months of enrollment the screen failure rate was amended to 22%. Total enrollment communicated with the regulatory bodies was 173 to account for screen failures, trial evaluation failures, and normal attrition [76/.71 = 108/.80 = 135/.78 = 173 (rounding up at each step)].

The noninferiority margin was set to preserve at least 50% of the expected effect [19, 20]. Assuming, based upon enrollment criteria and past literature [21], the average baseline pain is approximately 75 mm, a 30% improvement in VAS score would be 23 mm. The 7.5-mm margin on the VAS scale would preserve approximately 77% of the effect and would, therefore, be an appropriate margin.

#### Carryover estimations

Prior to analysis, measures will be inspected for carryover effects that may be present as a function of time and/or the order of therapy delivery. Carryover effects will be estimated using the within-subject sums of the score from week 1 to week 12 and from week 12 to week 24. An independent two-sided *t* test will test the null hypothesis of equal within-subject sums between the two sequence groups tonic/burst stimulation and burst/tonic stimulation. If the null hypothesis is not rejected at the 0.10 level, then the potential carryover effects are considered negligible. If the null hypothesis is rejected, the subsequent analysis will be carried out on the first-period data only.

#### Primary analysis

The primary effectiveness analysis is the 12-week noninferiority of the within-subject difference between tonic and burst stimulation for the mean daily overall VAS score calculated from the diary. The primary analysis will be conducted using the intention-to-treat population to include data recorded at the 12- and 24-week visits for all randomized participants, except as noted below. Missing data will be imputed using the hot-deck or last-observation-carried-forward method, as appropriate. If participants increase their pain medications during the first 24 weeks of the study, assessment of the effect of the therapy mode on pain will be confounded by the influence of additional pain medications. Thus, participants who increase pain medication (other than acetaminophen) after randomization will be considered treatment failures for the interval during which the increase occurred, and that participant’s baseline mean daily VAS score will be used for that interval in the analysis. A Z-statistic compared with the standard normal distribution will test the null hypothesis that the within-subject difference for the mean overall VAS score is less than or equal to 7.5 mm.

#### Additional analyses

For the control phase of the study, a responder analysis will inspect the proportion of participants achieving a change from baseline of 30% or greater for the overall VAS score during each stimulation mode. If noninferiority of burst stimulation is established in the primary endpoint analysis, the superiority of burst versus tonic stimulation will be tested for the overall VAS score using a one-sided *t* test. The within-subject difference for trunk and limb VAS scores will be inspected using two-sided 95% confidence intervals. The presence of paresthesiae will be compared between the two stimulation modes, and a relative difference of the within-subject experience will be computed. All other measures will be inspected using appropriate descriptive statistics and within-subject comparisons of burst versus tonic stimulation. Additional analyses will also include inspection of results in an intention-to-treat population to include all randomized participants; conservative imputations for missing data or participant withdrawal will be made in a manner appropriate to the measure and pattern of missing data.

During the open-label phase, type of therapy was chosen by the participant. Appropriate longitudinal statistics will examine long-term outcomes with the device- and participant-selected stimulation modes. Comparative analyses for burst versus tonic stimulation for this phase will be performed if statistically appropriate.

#### Safety

The safety profile of both stimulation modes will be characterized using appropriate descriptive statistics (e.g., incidence rates).

## Discussion

As the first large randomized controlled study of burst stimulation, the design is intended to provide evidence to support FDA approval of the burst neuromodulation waveform for the treatment of chronic neuropathic pain by establishing noninferiority of burst stimulation to traditional tonic stimulation. The results of this study will provide comparative efficacy and safety for burst stimulation in patients who are candidates for traditional SCS. Additionally, data from this study will provide evidence for the psychosocial and functional outcomes during both stimulation modes. The two phases of this study will facilitate comparisons between the waveforms as well as inspection of preference and long-term usage patterns for up to 2 years when the leads are placed for tonic stimulation. Chronic pain and responses to neurostimulation are both complex sensory phenomena. The ability to have patients experience the control and experimental stimulation modes is a unique feature of this study design, which should facilitate meaningful patient preference assessment.

The enrichment strategies for this study design confer advantages for our primary aim. The within-subject comparisons help to control variability in the data such that lead location, programming, and other participant-specific variables are held constant. Additionally, only participants who respond to tonic stimulation during the trial evaluation period and are candidates for a traditional SCS system are randomized, which should minimize participant attrition while facilitating comparative efficacy in patients who are known candidates for SCS. Ideal electrode placement for burst stimulation may not be at the site chosen for tonic stimulation, as in the present study. The same might be true of other waveforms; further study is required to address these points.

Furthermore, participants with marked depressive symptoms are excluded from eligibility in this study. Affective and functional dimensions are reflected in patients’ impression of change – even when a reduction in pain intensity may be considered clinically minimal [22]. Affective disruptions commonly associated with pain may also interfere with an accurate assessment of changes in pain intensity. Our study is intended to provide a comparative inspection of pain during burst and tonic stimulation that is largely independent of affective improvements that may indirectly influence participant’s assessment of therapy. We recognize that a minority of chronic pain patients, as few as 14% by some accounts, present without significant depressive symptoms [23, 24]. Excluding participants with depressive symptoms is not expected to limit generalizability, however, because the population included in this design is expected to provide a conservative estimate of pain relief during stimulation. Any direct or indirect benefits to affective function conferred by burst stimulation would be expected to be additive, not exclusive, to the effects on pain. Thus, the results of this study would be expected to approximate, possibly to a lesser degree, the efficacy of burst stimulation for general clinic populations.

In contrast, our sample of participants with minimal affective disruption is likely to create a “floor effect” whereby comparative improvements in psychosocial outcomes for burst versus tonic stimulation are unlikely to be detectable. Early evidence indicates that some psychosocial domains may be differentially affected during burst stimulation compared to tonic stimulation [5]; future studies will be needed to target this aspect of the therapy.

The cross-over design also imposes some limitations upon the hypotheses that can be addressed with these results. First, the presence of any carryover effects, although not expected, may limit our analysis by reducing the treatment window to the first 3-month period – thus leading to a between-subject comparison. Second, we will be unable to statistically assess changes in pain across time without losing the benefits of the within-subject design. Much of the literature for chronic pain treatments focuses on a calculated change in pain from baseline to follow-up. Our study design is not intended for such an analysis and any inspection of the similar results in our study will lead to limited conclusions, at best.

As with all studies, the design described herein is not without its limitations; however, this study represents the first large randomized controlled trial comparing the burst waveform with previously approved tonic waveforms. Ultimately, we expect to collect and report data leading to FDA approval for the device that can offer both burst and tonic waveforms, leading to individualized therapy strategies through enhanced therapeutic options.

## Trial status

The study is ongoing.

## Additional file

Additional file 1: SPIRIT Checklist. (PDF 130 kb)

## Abbreviations

AE(s): Adverse event(s)
BDI-II: Beck depression inventory version II
FDA: United States Food and Drug Administration
IDE: Investigational device exemption
IRB: Institutional review board
ODI: Oswestry disability index
PCS: Pain catastrophizing scale
PGIC: Patient global impression of change
SCS: Spinal cord stimulation
SF-36v2: Short Form 36 Health Survey version 2
SF-MPQ-2: Short Form McGill Pain Questionnaire version 2
VAS: Visual analog scale for pain
